# The genetic locus underlying red foliage and fruit skin traits is mapped to the same location in the two pear bud mutants ‘Red Zaosu’ and ‘Max Red Bartlett’

**DOI:** 10.1186/s41065-018-0063-7

**Published:** 2018-07-31

**Authors:** Huabai Xue, Suke Wang, Jia-Long Yao, Xiaoli Zhang, Jian Yang, Long Wang, Yanli Su, Lei Chen, Huirong Zhang, Xiugen Li

**Affiliations:** 1 Zhengzhou Fruit Research Institute, Chinese Academy of Agricultural Sciences, Zhengzhou, 450009 China; 2 The New Zealand Institute for Plant and Food Research Limited, 120 Mt Albert Road, Sandringham, Auckland, 1025 New Zealand; 3 Research Institute of Horticultural crops, Xinjiang Academy of Agricultural Sciences, Urumqi, 830091 China

**Keywords:** Bulk segregation analysis, Indel markers, *Pyrus*; red pear

## Abstract

**Background:**

Red-skinned pears are attractive to consumers because of their aesthetic appeal and the antioxidant-associated health benefits provided by the anthocyanins in their red skin. In China, the ‘Red Zaosu’ (RZS) red bud mutation of the Zaosu (ZS) pear has been used as a parent in Asian pear breeding to generate new cultivars with crispy red fruit and red tender shoots resembling those of the ‘Max Red Bartlett’ (MRB) pears.

**Results:**

In this study, a segregation ratio of 1:1 was observed between plants with red or green shoots in four families with RZS as the only red shoot gene donor parent, suggesting that the red shoot trait of RZS is associated with a dominant gene. Three markers, In1400–1, In1579–1 and In1579–3, were chosen from 22 pairs of indel primers targeting regions in the vicinity of the previously identified red fruit skin locus of MRB and were able to effectively distinguish the eight red shoot plants from the eight green shoot plants. Linkage analysis indicated that the genetic distance between the two marker loci (In1579–1 and In1579–3) and the red shoot locus of RZS were both 1.4 cM, while the genetic distance between the In1400–1 marker and the red shoot locus was 2.1 cM. The physical position of the red locus in RZS should be in the 368.6 kb candidate interval at the bottom of LG4.

**Conclusions:**

The genetic locus responsible for the red tender shoots of RZS was located in the same interval of the red fruit skin gene of MRB, meaning that the bud mutation loci of RZS and MRB may be the same or adjacent to each other, and the red shoot trait and the red fruit skin trait in RZS may be controlled by the same, or a closely linked locus. As a result, breeders could use red shoots as a morphological marker to select for the red-skinned hybrids from RZS families.

**Electronic supplementary material:**

The online version of this article (10.1186/s41065-018-0063-7) contains supplementary material, which is available to authorized users.

## Background

The pear (*Pyrus* spp.) is one of the main fruit trees in China and a popular fruit worldwide as a result of being nourishing, delicious and juicy. Pears are usually diploid with a genome size ranging from ~ 527 Mb [1] to ~ 600 Mb [2]. Pear skin color is either green, yellow, russet or red [3–5] with anthocyanin as the main component of red-skinned pears and the main determinant of the degree of red coloration [6, 7]. Anthocyanins contained in red-skinned pears consist of cyanidin-3-galactoside, peonidin-3-galactoside [8, 9], cyanidin-3-glucoside, cyanidin-3-arabinfuranoside and peonidin-3-glucoside [10, 11]. Red-skinned pears are favored by people because of their bright-colored appearance and the health benefits of anthocyanins [12–14].

The ‘Max Red Bartlett’ (MRB) pear, which has red tender shoots and fruits, is a red mutant cultivar of the famous European ‘Bartlett’ pear and is often used as a parent to breed new cultivars because of the strong inheritance of its bright red fruit skin [15, 16]. The red fruit skin trait of MRB is controlled by a single dominant gene called *Red*, which is located in the fourth linkage group (LG) [17]. The trait of red tender shoots in MRB correlates highly with the development of red fruit skin trait and are both inherited in the progeny, enabling the early identification of red-skinned fruit by their red tender shoots [18].

‘Zaosu’ (ZS) is a famous pear cultivar in China with green fruit skin and green shoots. ‘Red Zaosu’ (RZS), which has red tender shoots and fruits such as MRB, was a mutant of ZS found in Weibei, Shanxi province in China in 2004 [19]. We crossed RZS with pear cultivars that have green shoots and found that approximately half of the young seedlings had red tender shoots, indicating that the trait of red tender shoots in RZS was controlled by a dominant gene and could be inherited in its offspring. Other studies found that there might be a common mechanism of transcriptional regulation in the red fruit skin of RZS and MRB [20, 21], indicating that mutations in the same genetic locus may underlie red fruit skin and red shoot traits for RZS and MRB.

In pears, most of the structural and regulatory genes responsible for anthocyanin biosynthesis have been cloned and functionally characterized [22, 23]. In the two red-blushed Asian pear cultivars ‘Meirensu’ and ‘Yunhongli No.1’, phenylalanine ammonia-lyase (PAL) was found to participate in the first step of induced anthocyanin biosynthesis, while UDP-glucose: flavonoid-3-O-glucosyltransferase (UFGT) was closely related to anthocyanin accumulation [7]. In another study on two other red blushed pear cultivars ‘Mantianhong’ and ‘Aoguan’, PAL was not found to be a key enzyme for colouration, while the activity of chalcone isomerase (CHI) was found to be closely related to anthocyanin biosynthesis [24].

In the European pear ‘Early Red Doyenne du Comice’ and ‘Green Doyenne du Comice’, *PyMADS18* was found to participate in the regulation of anthocyanin synthesis during the early stages of fruit development [25]. MYB10 interacts with transcription factors bHLH and WD40 to form an MBW transcription complex that regulate gene transcription of anthocyanin biosynthesis and, consequently, anthocyanin accumulation [26, 27]. Further studies in pears showed that the expression level of *MYB10* was significantly higher in the full-red mutants (MRB and ‘Early Red Doyenne du Comice’compared) than in their green parents (‘Bartlett’ and ‘Green Doyenne du Comice’) at early stages of development [28, 29]. The demethylation and methylation of the *MYB10* (*PcMYB10* and *PyMYB10*) promoter might be associated with red-/green-skinned mutants [20, 21]. However, *PcMYB10* was located on LG9, but not LG4, of the *Red* locus and is unlikely to be directly responsible for the mutation of MRB [28].

There had been several reports regarding the transcriptomic and proteomic level describing the bud mutation mechanisms of RZS [22, 30, 31], but none have reported on the mapping of the bud mutation locus in RZS. This locus may play an important deterministic role in the red tender shoots and red fruit skin traits of RZS. In this study, we used the indel markers selected from the interval of the red fruit skin trait locus of MRB to map the red shoot trait locus of RZS using the segregation population of a cross between ‘PremP109’ and RZS. Our findings make it easier to recognize and understand the coloring mechanism of both the red traits of shoots and the red fruit skin color as well as the relationship between them.

## Methods

### Materials and methods

#### Plant materials

Four hybrid populations were used in this study to analyze the genetic regulation of the red tender shoot trait of RZS (Table 1). The seedlings of these populations were grown in a greenhouse for the first year and then transplanted to an experimental orchard in Xinxiang, Henan Province, or in Luntai, Xingjiang Province, China. ‘PremP109’ was a blushed pear cultivar with green tender shoots bred from oriental pears ‘Kousui’ and ‘Huoba’ by the New Zealand Plant & Food Research Institute (PFR), and whose parents were (Kousui×Huoba)^2^ and (Kousui×Huoba)^3^. Eight red shoot F1 individuals and eight green shoot plants were randomly selected from the progeny of a family of ‘PremP109’ × RZS cross and used to screen polymorphic primer pairs between red and green shoot plants. From the ‘PremP109’ and RZS cross, 144 progeny (72 plants with red tender shoots, and 72 with green tender shoots) were collected to be analyzed with the two parents to map the red shoot trait.

**Table 1:** Red shoot color segregation ratios in four families with ‘Red Zaosu’ as a parent

Year of cross	Female parent	Male parent	Observed ratio (seedlings, Red:Green)	χ^2^ (Red:Green | 1:1)
2015	‘PremP109’ (Red-blushed skin, green shoots)	‘Red Zaosu’ (Full-red skin, red shoots)	128:106	2.068
2015	‘Korla pear’ (Red-blushed skin, green shoots)	‘Red Zaosu’ (Full-red skin, red shoots)	906:805	5.962^a^
2014	‘Whangkeumbae’ (Ruset skin, green shoots)	‘Red Zaosu’ (Full-red skin, red shoots)	54:68	1.607
2014	‘Red Zaosu’ (Full-red skin, red shoots)	‘Hongbaoshi’ (Red-blushed skin, green shoots)	60:62	0.033

χ^2^ test for segregation: ^a^ *P* < 5%

#### DNA extraction

Young leaves of two parents and 144 seedlings from the ‘PremP109’ × RZS cross were collected in April 2017. The leaves were immediately frozen in liquid nitrogen and transferred to a − 80 °C freezer. Genomic DNA of the young leaves was extracted using a combination of the cetyltrimethylammonium bromide (CTAB) method and a centrifugal column [32]. DNA integrity and quality were detected by 1% agarose gel electrophoresis, and NanoDrop (Thermo, USA). The DNA samples were diluted to 20 ng/μL with sterile ultrapure water and stored at − 20 °C for later use.

#### Linkage group comparison

The previously published SNP and SSR high density linkage map of ‘Bayuehong’ × ‘Dangshansu’ [33] was used as a reference. The LG4 of ‘Abbe Fetel’ × ‘Max Red Bartlett’, which contains the red-skinned trait loci *Red* [17], was aligned to the reference map by two common SSR markers. A comparison linkage map was then constructed using the MapChart software [34]. The genetic positions of two common SSR markers (CH02c02 and CH01d03) and the Red locus on LG4 of ‘Abbé Fétel’ × MRB and ‘Bayuehong’ × ‘Dangshansuli’ could be considered as a geometric sequence, and the genetic position of the *Red* locus in the genetic map of ‘Bayuehong’ × ‘Dangshansu’ could be estimated according to its relative position to the two common SSR markers on the two LGs. Additionally, the physical interval of the *Red* locus could also be estimated according to physical positions of the SNP markers (CTG1064355 and CN900214) flanking the locus.

#### Primers selection

According to the results of the linkage group comparison, the general physical interval of the *Red* gene was estimated, and the nearby corresponding SNP loci (CTG1064355 and CN900214) on LG4 of the ‘Bayuehong’ × ‘Dangshansuli’ map were matched to the corresponding genomic intervals. The indel markers in the nearby interval of the *Red* gene locus were selected from the previously constructed indel marker database (http://genedenovoweb.ticp.net:81/pear/) for subsequent use. The indel database, which covers the whole pear genome, was previously developed by re-sequencing 17 red and non-red pear varieties (data not published).

#### Preliminary screening of primers

Primers were preliminarily screened using 8 red shoot individuals and 8 green shoot individuals from the ‘PremP109’ × RZS cross. Each sample was run in 20 μL of PCR reaction mixture containing 1× reaction buffer (Mg ^2+^ plus), 0.2 mM dNTPs, 0.5 U of Taq polymerase (TaKaRa, Dalian), 0.3 μM each primer, and 20 ng of genomic DNA. The PCR conditions consisted of the following: pre-denaturation (98 °C for 20 s) followed by 29 cycles of denaturation (98 °C for 10 s), annealing (55 °C for 30 s), extension (72 °C for 45 s), and a final extension cycle (72 °C for 10 min).

#### Linkage analysis

DNA markers were amplified from two parents and 144 hybrids using indel primers linked to the red shoot trait. According to the theory of double false test cross proposed by Weeden et al. (1994) [35] and using the JoinMap4.0 software [36], band types of all DNA markers detected in the population were classified into five types of separation modes: lm × ll, nn × np, hk × hk, ef × eg and ab × cd. Bands that did not amplify or that were unclear were marked as “--”. Red and green shoot traits were defined as phenotypic markers. The Kosambi mapping function of the JoinMap4.0 software was used to calculate genetic distance between markers and traits. A linkage map of markers and traits was drawn using the MapChart 2.30 software [34].

## Results

### The red shoot trait of RZS was controlled by a dominant genetic locus

**Fig. 1:**
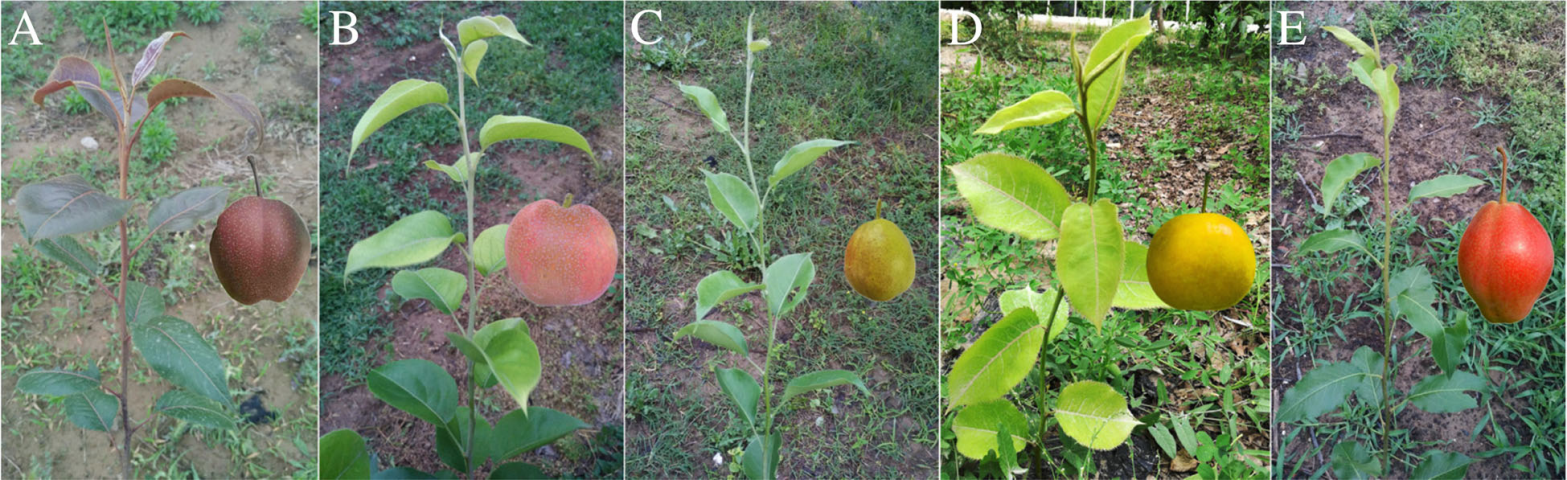
New shoots and fruits of the parents of the four families. **a**: ‘Red Zaosu’, **b**: ‘PremP109’, **c**: ‘Korla pear’, **d**: ‘Whangkeumbae’, **e**: ‘Hongbaoshi’

Four families derived from crosses between RZS and a green shoot parent were used in segregation analyses (Table 1 & Fig. 1). Three of the four families showed a red: green segregation ratio that was not significantly different from a 1:1 ratio. In contrast, the ratio for the family ‘Korla pear’ × RZS was not significantly different from a green: red = 1:1 ratio (*P* < 5%). The 1:1 segregation ratio of the red shoot trait in three RZS families indicated that the red shoot trait in RZS was controlled by one dominant gene.

### Linkage group comparison

**Fig. 2:**
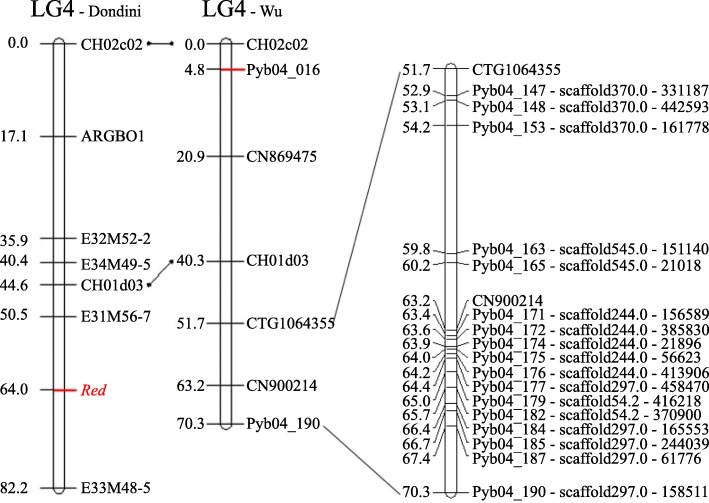
Comparison between Dondini’s LG4 and Wu’s LG4. The left one shows the *Red* locus on LG4 in Dondini’s map (2008), the middle one is the simplified LG4 of Wu’s high density genetic map (2014), the right one was part of LG4 of Wu’s map that might contain the *Red* locus interval we estimated**.** The middle and the right maps were drawed according to the supplementary data of Wu’s article, and the physical positions of the SNP markers were also added to themselves.

Results of the linkage group comparisons indicated that the SSR markers CH02c02 and CH01d03 located on the LG4 of ‘Abbé Fétel’ × MRB [17] were also located on the LG4 of ‘Bayuehong’ × ‘Dangshansuli’ [33]. The order and location of the two markers are similar between the two linkage groups, indicating that the two linkage groups were of the same linkage group (Fig. 2). Supposing that genetic positions of CH02c02 and CH01d03 and the *Red* locus on the two LGs represent a geometric sequence, the *Red* locus was estimated to be located at around the 57.8 cM ($\frac{64.0}{44.6}\times 40.3$=57.8) on the LG4 of ‘Bayuehong’ × ‘Dangshansuli’ from Fig. 2 [33], indicating that it might be located within the 51.7 cM ~ 70.3 cM interval according to the markers CTG1064355 and CN900214. Since the estimated position of the *Red* locus and the position of marker CN900214 were too close to resolve, we defined the region between markers CTG1064355 and Pyb04_190 as the candidate interval of the *Red* gene.

### Primer selection

SNP loci within the 51.7 cM ~ 70.3 cM interval on the LG4 of ‘Bayuehong’ × ‘Dangshansuli’ (Fig. 2) were mapped to the scaffolds where they were from according to their physical positions. Twenty-two pairs of primers from scaffolds within the interval were selected from the indel marker database (http://genedenovoweb.ticp.net:81/pear/), which was built by our team for subsequent use. The specific primer information is shown in Table 2. The nomenclature of the scaffolds used in Wu’s article and the marker database were confirmed and matched according to the file ‘Scaffold_names’ in the FTP space of the reference genome ‘Dangshansuli’.

**Table 2:** Information of 22 pairs of specific indel primers used in this study

Primer	Scaffold	Position	Forward primer	Reverse primer
In1270–1	NW_008988270.1 (Scaffold 244.0)	65,865	AGAAAGGAGGAGGAGGAAG	AAATCCAAGCCCTATAAACC
In1270–2	NW_008988270.1 (Scaffold 244.0)	128,003	AATAGGCCAGCATACCATAA	TAATGAACAATACCCATCCG
In1270–3	NW_008988270.1 (Scaffold 244.0)	160,055	ATTGCTACAAAGCTGCTCTC	TATTTTGCCCTTACAGTTCG
In1270–4	NW_008988270.1 (Scaffold 244.0)	208,561	TACCTACCGGTGTCTGATTC	ATTGTTTTGGTTTTGTTGCT
In1270–5	NW_008988270.1 (Scaffold 244.0)	257,130	TTTTAAGGGGCAATTTATGA	AACCTCCAAAAACAAACAAA
In1270–6	NW_008988270.1 (Scaffold 244.0)	309,525	AGAATTCAGAAATGGGGTTT	TAATCGACATTGACACGAAA
In1270–7	NW_008988270.1 (Scaffold 244.0)	352,640	CGTGAGAGCTTACTCAGACC	CTAATGTTATTGAAGCGGGT
In1270–8	NW_008988270.1 (Scaffold 244.0)	403,691	ATAGAACGGGTCTTTTTGGT	TTTCATAATTTGTGGGACATT
In1270–9	NW_008988270.1 (Scaffold 244.0)	457,893	TCTAAGGGCTGGACAGAATA	TTAAGTAAGGAGTCGGCAAC
In1322–1	NW_008988322.1 (Scaffold 297.0)	125,085	TTGAGGTTTCTTTTTGGTTG	TTGATATAAACCAGGGGATG
In1322–2	NW_008988322.1 (Scaffold 297.0)	150,815	TTACTGACAGCATCTGACCA	CAACTGGATGATTTCCTCAT
In1322–3	NW_008988322.1 (Scaffold 297.0)	202,780	GCCTTTCTGTTTGCTAGAAG	ATCTTCTACAACTTGCTGCC
In1322–4	NW_008988322.1 (Scaffold 297.0)	250,555	TCTAAAAATGAAGCAGACCC	CTCTTTCGATTTTCTTGTGC
In1400–1	NW_008988400.1 (Scaffold370.0)	161,533	CAAGGACCAAAGTCACGTAT	TCTTCGACTTTAGGAAAAGAGT
In1400–2	NW_008988400.1 (Scaffold370.0)	204,789	GTATTTATGGACAAGCAGGC	TAACCACCCTGAGAATATGG
In1400–3	NW_008988400.1 (Scaffold370.0)	244,039	AAAATCGTTCCTTTCATGG	GAGGTTAAGCCCACTCCTAT
In1400–4	NW_008988400.1 (Scaffold370.0)	305,894	GAAATGAAAGAACGAAGGTG	TTTGACTTTTCTTCTGTGGG
In1400–5	NW_008988400.1 (Scaffold370.0)	344,900	AAGAAAAAGGGGCTTTTAGA	AATCCATTCGGTACAGTCAG
In1579–1	NW_008988579.1 (Scaffold545.0)	23,081	CATGTTACAGGTCCAACCTT	CCTATTGCAATCTGAAATCC
In1579–2	NW_008988579.1 (Scaffold545.0)	50,472	GCCCTAATTAAATGTCCTCA	AGGTGAGATCACAAGTGGAC
In1579–3	NW_008988579.1 (Scaffold545.0)	100,223	TTTCGACTCTTGCTTACCTC	ACGAAGTGCTTTTTACCAAA
In1579–4	NW_008988579.1 (Scaffold545.0)	152,991	GGAGTCTGGCTCATGTAATC	ACTTGGGCTATAGGGACACT

### Preliminary screening of primers and linkage analysis

**Fig. 3:**
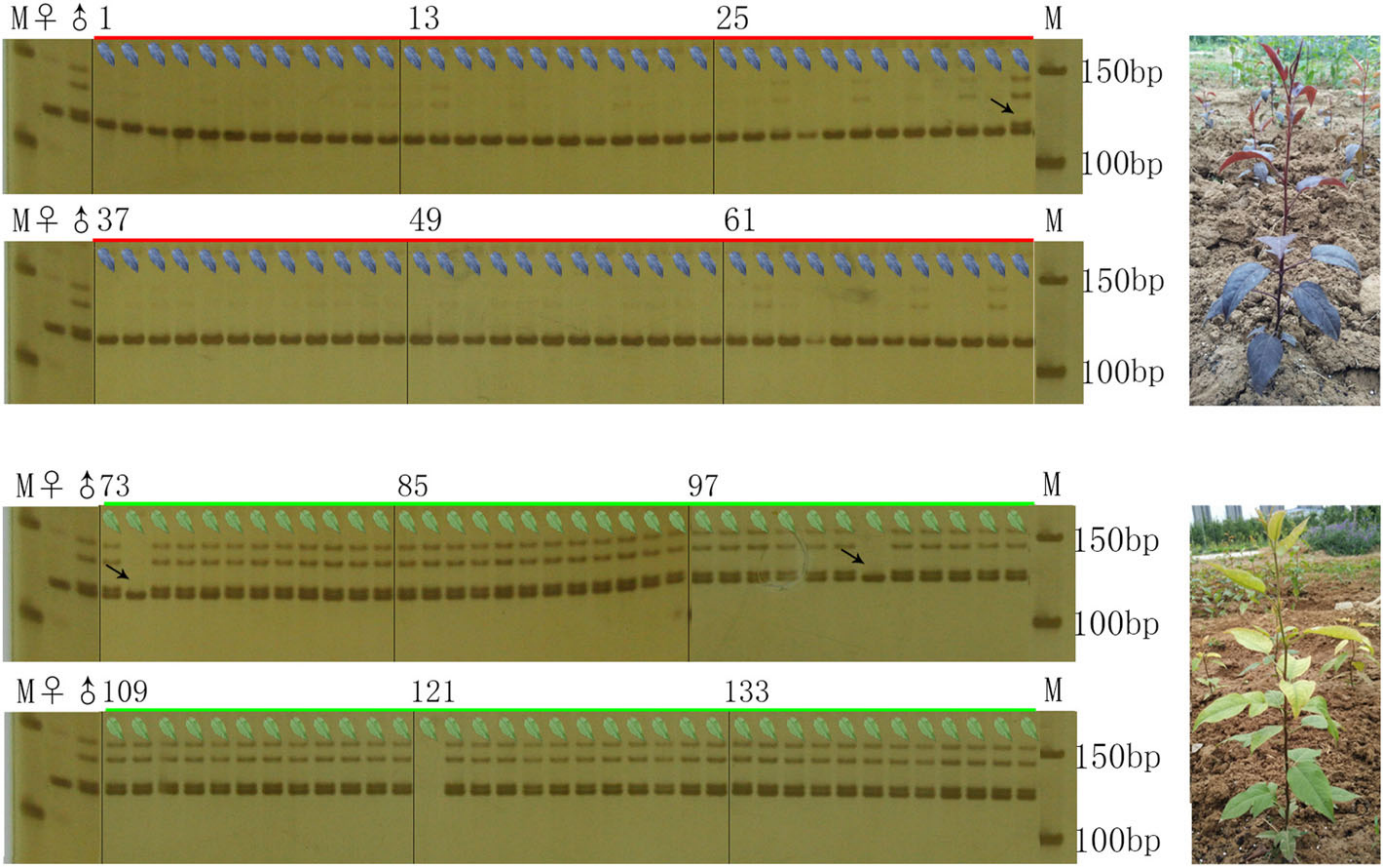
Amplification results using primers for the marker In1400–1 from the progeny plants of ‘PremP109’ × ‘Red Zaosu’. M: Marker; ♀: ‘PremP109’; ♂: ‘Red Zaosu’; 1–72: Red seedlings; 73–144: Green seedlings; Right pictures were red and green seedlings; Arrows in the figure refer to the exchanging seedlings.

Twenty-two indel primers were used in preliminary screening of the 8 red shoot and 8 green shoot seedlings of ‘PremP109’ × RZS. Three markers, In1400–1, In1579–1 and In1579–3, could distinguish red from green hybrid plants. This result suggested that these three markers and the red shoot trait in RZS were in linkage disequilibrium, and the three markers were further used to test 144 hybrids (Fig. 3).

**Fig. 4:**
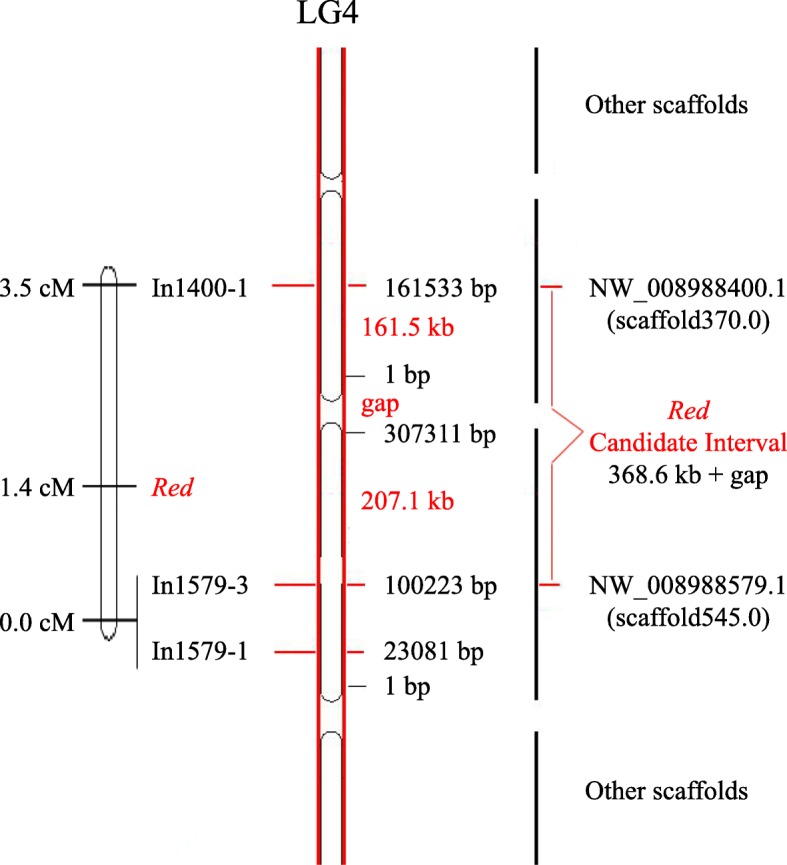
Molecular markers and the *Red* locus on LG4. The left panel shows the result of linkage mapping and the position of *Red* locus. The middle panel shows the reference genome assembly of LG4. The black vertical bar on the right panel indicates the corresponding positions of the scaffolds on the chromosome map, and the red line on the right indicates the mapping interval for the *Red* locus candidate genes.

A 3.5 cM linkage group, including the molecular markers and the red shoot trait locus in RZS, was identified, as shown in Fig. 4. The RZS red trait locus (*Red*) was located at 1.4 cM in the linkage map, and the rest of the markers were located at the opposite ends of the linkage group. The markers In1579–1 and In1579–3 were closest to *Red* at a distance of 1.4 cM, while the marker In1400–1 was farthest from *Red* at a distance of 2.1 cM.

The *Red* locus in RZS was located between marker In1400–1, which was identified in scaffolds NW_008988400.1 (scaffold370.0), while markers In1579–3 and In1579–1, both belonged to NW_008988579.1 (scaffold545.0). These two scaffolds neighbored each other on the bottom of LG4. Therefore, the *Red* loci of RZS should be in the 368.6 kb candidate interval at the bottom of LG4, and the target candidate interval can be further divided into two subintervals: a 207.1 kb interval from 100,223 bp to 307,311 bp in scaffold NW_008988579.1, and a 161.5 kb interval from 1 bp to 161,533 bp in scaffold NW_008988400.1. It is worth noting that there is a gap between scaffolds NW_008988579.1 and NW_008988400.1 and that the size of the gap is unknown, which could change the size of the map intervals for the *Red* locus (Fig. 4).

Based on the reference pear genome, 28 gene models were predicted in the candidate 368.6 kb genomic region (Additional file 1: TableS1). Among these, 22 have a predictive gene function. However, structural genes associated with anthocyanin biosynthesis such as *PAL*, *CHS*, *CHI*, *DFR* (dihydroflavonol 4-reductase), *ANS* and *UFGT* or the regulatory gene MYB10 transcription factor were not found within the candidate region.

## Discussion

China is the origin of oriental pears and has an abundant resource of pear species and cultivars [37]. These pears include some germplasms with red-blushed fruit belonging to commonly cultivated species, such as *P. pyrifolia* sand pear group, *P. pyrifolia* white pear group, *P. ussuriensis* Maxim and *P. Sinkiangensis* Yu, although the amounts of these types of germplasms are relatively small in China [4, 5, 19, 38]. Using these germplasms, Chinese pear breeders have bred blushed pear cultivars such as ‘Hongxiangsu’ [39], ‘Yuluxiang’ [40], ‘Hanhongli’ [41] and ‘Xinli No.7’ [42]. These pear cultivars are praised by consumers due to their bright appearance, yet full-red varieties resembling European pears MRB and ‘Red Clapp’s Favorite’ (RCF) are relatively infrequent in oriental pears [32]. To address this, resources should be introduced for pear breeders in China to develop full-red colored Asian pear cultivars. The fruit skin color, period of fruit skin coloration and tender shoot color in RZS are similar to those in MRB. Additionally, the inheritance of the red shoot trait of RZS was also consistent with that of MRB according to the 1:1 segregation ratio in the four RZS families included in this study. The crisp RZS was very similar to the European MRB parent on their red traits, indicating that it could be a valuable genetic resource for breeding full-red colored and crisp Asian pears. This is essential because most of the offspring derived from crosses between crisp Asian pears and soft European pears produce fruits with a soft flesh texture [43, 44]. In addition, it is rare for pear trees with white flowers and red shoots to be used as ornamental fruiting plants, and RZS can also be employed to breed new ornamental cultivars displaying white flowers, red fruit and red shoots, resembling the red leaf peach variety ‘Hongyetao’ [45].

The traits of red skin and red shoots are co-segregated in both RZS and MRB [15, 16, 46]. These similarities suggest that the same or a similar bud mutation is responsible for the red color traits in both RZS and MRB. RZS is a red bud mutant of ZS, a cultivar derived from a cross between the ‘Pingguoli’ pear and the ‘Shenbuzhi’ pear, which is an offspring of an unknown European pear and an unknown Asian pear. However, it is unclear whether the part of the RZS genome that is generated from the ZS mutation is derived from the genetic composition of an unknown European pear. In this study, the red shoot trait in RZS was studied by using indel markers located next to the red skin locus *Red* in MRB. The results showed that the genomic variation loci of RZS derived from ZS may be the same or closely linked to the variation loci of MRB that are derived from ‘Bartlett’ pear. These findings also confirmed that MRB was phenotypically very similar to RZS at a genomic level. On the other hand, these findings indicate that the red shoot trait in RZS may be controlled by the same locus as the red skin trait or by a closely linked locus. Therefore, the red shoot trait of the offspring of RZS may be used as a phenotypic marker of the red skin trait for the initial selection of red-skinned offspring.

In a previous study, we performed QTL-seq analysis on red- and green- pools derived from a red hybrid population resulting from a ‘Mantianhong’ × ‘Hongxiangsu’ cross and mapped the red-blushed skin trait locus of the Asian pear to the fifth linkage group [47]. Yao et al. similarly positioned the red-blushed skin trait locus of the European pear in the same interval as the Asian pear using a hybrid population resulting from a ‘Bayuehong’ × ‘Dangshansuli’ cross [33, 48]. This shows that Asian and European pears may share the same coloring mechanism, which results in red-blushed fruit skin. In the bud mutants with red shoots and full-red fruit skin, we demonstrated that the inheritance patterns and the loci of the red traits of RZS might be the same as those of the European pear mutant MRB [17, 18]. RCF, a full-red mutant of the European pear cultivar ‘Clapp’s Favorite’, is similar to MRB and RZS with a red fully colored fruit skin during its early fruiting period, yet unlike MRB and RZS, its shoots are green. Interestingly, our research on trait mapping a hybrid population derived from a cross of RCF and the Japanese pear cultivar ‘Mansoo’ showed that the red skin trait loci of RCF were located on LG5 (not LG4), which was similar to the trait loci of red-blushed pears but different from those of MRB and RZS (data are in submission). Taken together, the existing research indicates that red pears can be roughly divided into three sub-types: the red-blushed type, the full-red mutant with red shoots like the MRB pear, and the full-red mutant with green shoots like the RCF pear. For the two types of red mutants, further research is required to identify the differences between their mutation sites and their coloring mechanisms. Additionally, research of the color traits of their new shoots may provide a unique entry point for related works.

In this study, we mapped the red shoot trait of RZS to the same region as the full-red fruit skin trait gene identified in a previous study of the MRB [17]. The candidate interval located on LG4 narrowed the search for candidate genes to a smaller range. The LG4 region contains a gap of unknown size and a known 368.6-kb region consisting of parts of the two scaffolds, NW_008988400.1 and NW_008988579.1. However, there were no structural and regulatory genes associated with anthocyanin biosynthesis and accumulation were found in the known 368.6-kb region. The length of the two scaffolds (NW_008988400.1 and NW_008988579.1) were only 446,383 bp and 307,311 bp, respectively, but there were only a few markers linked to the red shoot trait gene in RZS. The lack of a large number of molecular markers tightly linked to the target gene locus indicates that the actual physical distance between the markers and the target locus is relatively far, and the gap might be much more large than the known 368.6 kb region. Although there is a histone-lysine N-methyltransferase ATX2-like gene related to demethylation, found in the 368.6 kb candidate region of the *Red* gene in this study, that might be related to the regulation of anthocyanin biosynthesis and accumulation according to previous studies [20, 21], it was probably not the *Red* gene itself due to the relatively long distance between it and the *Red* locus. Presently, we cannot identify more scaffolds within the gap that would further narrow the *Red* gene candidate region according to the high-density genetic map [33], while fine-mapping and functional identification of the *Red* gene requires more efforts, much more work, and a better assembly for the pear reference genome.

## Conclusion

Red tender shoot color was controlled by a single dominant gene in four RZS families as indicated by a 1:1 (red: green) segregation ratio. Three indel markers that were strongly linked to the previously published red fruit skin gene locus of MRB were also closely linked to the red shoot locus in RZS. The locus was mapped to a 368.6 kb candidate interval (with a gap of unknown size) on LG4 of RZS. These results indicate that the bud mutation of RZS and MRB may result from the same locus or from adjacent loci, and these regions have a decisive role in the fruit skin color and shoot color. Breeders could use the red shoots as a phenotypic marker to select for the red-skinned hybrids from RZS families.

## Additional files

Additional file 1: **Table S1.** 28 gene models predicted in the candidate 368.6 kb genomic region (XLS 25 kb).

## Abbreviations

ANS: Anthocyanidin synthase
CHI: Chalcone isomerase
CHS: Chalcone synthase
CTAB: Cetyltrimethylammonium Bromide
DFR: Dihydroflavonol 4-Reductase
F3H: Flavanone 3-hydroxylas
InDel: Insertion/Deletion
LG: Linkage Group
MRB: Max Red Bartlett
PAL: Phenylalnine Ammonia Lyase
PCD: programmed cell death
RCF: Red Clapp’s Favorite
RZS: Red Zaosu
SNP: single nucleotide polymorphism
UFGT: UDP-glucose:flavonoid 3-O-glucosyltransferase
ZS: Zaosu
